# Determinants of access to healthcare by older persons in Uganda: a cross-sectional study

**DOI:** 10.1186/s12939-015-0157-z

**Published:** 2015-03-07

**Authors:** Stephen Ojiambo Wandera, Betty Kwagala, James Ntozi

**Affiliations:** Department of Population Studies, School of Statistics and Planning, College of Business and Management Sciences, Makerere University, P.O. Box 7062, Kampala, Uganda; Consortium for Advanced Research Training in Africa (CARTA cohort 2) Fellow at the African Population and Health Research Centre (APHRC), Nairobi, Kenya

**Keywords:** Africa, Uganda, Utilization, Health care, Health services, Elderly

## Abstract

**Background:**

Older persons report poor health status and greater need for healthcare. However, there is limited research on older persons’ healthcare disparities in Uganda. Therefore, this paper aimed at investigating factors associated with older persons’ healthcare access in Uganda, using a nationally representative sample.

**Methods:**

We conducted secondary analysis of data from a sample of 1602 older persons who reported being sick in the last 30 days preceding the Uganda National Household Survey. We used frequency distributions for descriptive data analysis and chi-square tests to identify initial associations. We fit generalized linear models (GLM) with the poisson family and the log link function, to obtain incidence risk ratios (RR) of accessing healthcare in the last 30 days, by older persons in Uganda.

**Results:**

More than three quarters (76%) of the older persons accessed healthcare in the last 30 days. Access to healthcare in the last 30 days was reduced for older persons from poor households (RR = 0.91, 95% CI: 0.83-0.99); with some walking difficulty (RR = 0.90, 95% CI: 0.83-0.97); or with a lot of walking difficulty (RR = 0.84, 95% CI: 0.75-0.95). Conversely, accessing healthcare in the last 30 days for older persons increased for those who earned wages (RR = 1.08, 95% CI: 1.00-1.15) and missed work due to illness for 1–7 days (RR = 1.19, 95% CI: 1.10-1.30); and 8–14 days (RR = 1.19, 95% CI: 1.07-1.31). In addition, those who reported non-communicable diseases (NCDs) such as heart disease, hypertension or diabetes (RR = 1.09, 95% CI: 1.01-1.16); were more likely to access healthcare during the last 30 days.

**Conclusion:**

In the Ugandan context, health need factors (self-reported NCDs, severity of illness and mobility limitations) and enabling factors (household wealth status and earning wages in particular) were the most important determinants of accessing healthcare in the last 30 days among older persons.

**Electronic supplementary material:**

The online version of this article (doi:10.1186/s12939-015-0157-z) contains supplementary material, which is available to authorized users.

## Introduction

Population ageing is a global demographic event of the 21st century [1]. Worldwide, the proportion of older persons (age 60 and older) is 11% and shall increase to 20% by 2050. Similarly, the absolute number of older people has risen from 205 million in 1950 to 810 million in 2012 and shall increase further to 1 billion by 2022 and to 2 billion by 2050 – outnumbering children aged 0–14 years [2,3]. In sub-Saharan Africa (SSA), the absolute numbers of older persons (aged 60+) is 43 million (5% of the population) [4]. This shall increase to 163 million (8.3% of the population) by 2050 [3]. In Uganda, the number of older persons has increased from 1.1 million in 2002 (4.5% of the population) to 1.3 million in 2010 [5] and shall increase to 5.5 million (5.7% of the population) by 2050 [3].

There is no “standard numerical criterion for defining old age” [1], though age 60 and older, is used by both the United Nations (UN), the African Union [6,7] and the Ministry of Gender, Labour and Social Development (MoGLSD) in Uganda [5,8]. However, this 60+ age cut-off is inappropriate in many African contexts given the lower life expectancy [1]. Therefore, the World Health Organization set age 50 and older, as the cut –off to refer to the older persons in Africa [1,6]. Consequently, several studies have used this definition in Kenya [9,10]; Uganda [11] and South Africa [12]. Similarly, this paper used age 50 and older to define older persons.

Ageing and health is a growing concern in the discourse on ageing in SSA. Two major concerns emerge: first, the “vulnerability of older persons to detrimental health outcomes” [13] including high prevalence of NCDs and disabilities. The second is the limited access to healthcare [4,13-15].

In SSA, WHO SAGE studies and the IN-DEPTH network studies [16-20] have investigated older persons’ health status. Studies from the IN-DEPTH sites include South Africa [17]; Tanzania [20]; Vietnam [21]; Uganda [22]; Indonesia [23] and Bangladesh [24]. However, few have investigated healthcare access inequalities among older persons [1]. Those that have attempted to address healthcare inequalities have not nationally representative samples.

In Uganda, in the past decade, few studies addressed older people’s health needs. Available research has focused on HIV/AIDS in old age: anti-retroviral treatment [11]; and caregiving roles of older persons [25-28]. Other studies investigated older persons’ vulnerabilities [29]. These studies have underscored the presence of major unmet healthcare needs among Uganda’s older persons [22,30]. Those that have attempted to address access to healthcare were based on qualitative data and specific interest groups like the diabetics and not necessarily the older population [15,31]. There is urgent need for robust and nationally representative evidence on the inequalities in healthcare access among older persons [13]. Therefore, this paper aimed at examining the inequalities in healthcare access among older persons in Uganda, using a nationally representative sample.

## Methods

### Data source

This paper used the 2010 Uganda National Household Survey (UNHS) data with permission from the Uganda Bureau of Statistics. The UNHS was a cross sectional survey that covered individual and household characteristics including demographic and socio-economic characteristics, disability, self-reported NCDs and health seeking behavior of individuals. The description of the sampling procedures for the 2010 UNHS are described elsewhere [5]. A total of 6,800 households were interviewed in the survey [5].

Selection of older persons from the UNHS data used two variables: age and illness status. First, we selected older persons aged 50 years and older. This gave us an unweighted sample of 2,628 older persons. Second, we selected those who were sick in the last 30 days preceding the survey using the question: “During the past 30 days, did you suffer from any illness or injury?” (coded as 1 = Yes and 2 = No). The unweighted and weighted sample sizes of older persons were 1602 and 1478 respectively. We weighted the data to account for survey design, clustering and stratification effects.

### Measures of outcome variable

In the UNHS, the outcome variable, access to healthcare in the last 30 days, was measured as the “choice of service provider for use of health services” or consultation [32]. The survey had two questions on healthcare: 1) Did you consult any one during a major illness or injury in the last 30 days preceding the survey? This question was binary measure (yes and no). Those who consulted with any one were asked a follow up question: 2) Where did you go for consultation? (Additional file 1).

As shown in Table 1, the place of consultation were: drugs at home; neighbor/friend; community health worker; homapak drug distributor; ordinary shop; drug shop/pharmacy; private clinic; health unit government; health unit NGO; hospital government; hospital NGO; traditional healer; and others.

**Table 1:** **Choice of provider for health services use**

	**Where did you go for the first consultation during the past 30 days**		
	**Categories**	**Frequency**	**Percent (%)**
Did not access healthcare (1–5)	1 = Drugs at Home	32	2.4
	2 = Neighbor/Friend	5	0.4
	3 = Community health worker	12	0.9
	4 = Homapak drug distributor	1	0.1
	5 = Ordinary shop	13	1.0
Accessed healthcare (6–11)	6 = Drug shop/Pharmacy	134	10.1
	7 = Private clinic	408	30.9
	8 = Health unit government	414	31.3
	9 = Health unit NGO	48	3.6
	10 = Hospital government	160	12.1
	11 = Hospital NGO	54	4.1
Did not access healthcare (12, 96)	12 = Traditional healer	25	1.9
	96 = Other (specify)	15	1.1
	Total	1,321	100

281 did not access any care, making a total of 1602 older persons (unweighted sample).

In this paper, accessing healthcare in the last 30 days was recoded as a binary outcome (0 = No and 1 = Yes). Accessing healthcare in the last 30 days meant visiting formal health facilities either: public health facilities (government health unit and hospital); or private health facilities. Private facilities included private not for profit (PNFP) health facilities (NGO health unit and hospital); and Private for Profit (PFP) facilities (private clinics/drug shops or pharmacies). This categorization has been used in a Ugandan study on access to healthcare, though not focused on older persons [32].

Those who did not consult any one or used drugs at home, from a neighbor, visited a community health worker/homapak distributer, bought drugs from ordinary shop, and visited traditional healers and other places, were not considered to have accessed formal healthcare in the last 30 days. This exclusion was because Uganda’s formal healthcare is delivered through public health facilities and private facilities (both PNFP and PFP facilities) as reported elsewhere [32]. In addition, community health workers, homapak drug distributors and traditional healers were few in number (see Table 1) and their inclusion could not make a significant contribution to the study.

### Measures of explanatory variables

Using the Andersen’s behavioral model of health services use [33,34], explanatory variables were grouped into four categories: predisposing (demographic and socio-economic factors), health need and enabling factors. Demographic and socio-economic factors included: age, gender, marital status, religion, place of residence and living arrangements. Age was recoded into four age groups: 50–59, 60–69, 70–79 and 80+. Marital status was recoded as married, separated/divorced and widowed. Religion was categorized into Anglican, Catholic, Pentecostal, Moslem and others. Place of residence was dichotomized into urban and rural categories. Gender was coded as male and female. Living arrangements was recoded into two categories: either living alone or with others. Health need factors included disability status (1 = yes, 0 = no), self-reported NCDs (heart disease, diabetes and hypertension) and tobacco smoking. Enabling factors included education level, household poverty status (1 = poor, 0 = not poor, based on spending <1 dollar a day definition), and household assets such as ownership of a bicycle and land.

### Statistical analyses

We present descriptive statistics in form of frequency distributions. We used Pearson chi-square tests for the associations between socio-demographic, need and enabling factors and access to healthcare in the last 30 days. We chose 5% as the level of statistical significance. Disease duration was excluded from the analysis because it was highly correlated with disease severity (correlation coefficient = 0.44). A scatter plot of the two variables showed a strong positive linear relationship between the two variables [results not presented]. Removing diseases duration removed collinearity.

Finally, we fit Generalized Linear Models (GLM) with the Poisson family and the log link, while adjusting for survey design to obtain incidence Risk Ratios (RR) of accessing healthcare in the last 30 days, by older persons in Uganda. This was because the outcome variable was common and dichotomized [35-38]. In addition, although the GLM with the binomial family and log link regression was possible, we did not use it because of convergence problems during the estimation process. We weighted the analysis (using survey (svy) commands) to account for the survey design including clustering, and stratification. We analyzed the data using STATA version 13.

## Results

### Distribution of sick older persons by demographic and socio-economic factors

From Table 2, more than half of the respondents were women and majority were aged 50–59. Close to a third were from the eastern region. Most of the older persons were rural residents, lived with other people and headed households. More than half were married and had no formal education. In terms of religion, majority were Catholics followed by Anglicans. The majority were from non-poor households. Majority belonged to households that owned land, did not own a bicycle and depended on farming as a major source of earning.

**Table 2:** **Distribution of sick older persons by demographic, socio-economic factors and access to healthcare in Uganda**

	**Percent (%)**	**Frequency**	**% Accessed healthcare**	**p-value**
**Gender**				0.304
Female	56.6	837	74.6	
Male	43.4	641	77.1	
**Age group**				0.000
50-59	40.2	594	78.9	
60-69	28.1	416	80.7	
70-79	21.2	313	69.7	
80+	10.5	155	62.0	
**Region**				0.055
Central	24.0	354	72.6	
Eastern	32.9	486	78.7	
Northern	19.4	287	78.9	
Western	23.7	350	71.9	
**Place of residence**				0.129
Rural	91.8	1357	75.1	
Urban	8.2	121	82.2	
**Living alone**				0.003
No	89.4	1321	76.9	
Yes	10.6	157	65.3	
**Relationship to household head**				0.383
Head	71.6	1058	76.1	
Spouse	18.8	277	76.7	
Relative	9.6	143	70.8	
**Marital status**				0.027
Married	55.2	815	78.6	
Divorced/separated	10.7	158	71.8	
Widowed	34.1	504	72.3	
**Education level**				0.346
None	68.3	1009	76.4	
Primary	24.6	363	72.8	
Secondary or higher	7.1	105	78.5	
**Religion**				0.513
Catholic	46.0	680	74.2	
Anglican	35.1	519	77.1	
Muslim	9.5	140	81.1	
Pentecostal	7.0	103	72.1	
Seventh Day Adventists & others	2.4	36	72.8	
**Poverty status**				0.004
Non-poor	77.6	1146	77.5	
Poor	22.4	331	69.4	
**Household owns land**				0.476
No	10.8	160	73.2	
Yes	89.2	1318	76.0	
**Household owns bicycle as transport means**				0.001
No	62.8	928	72.8	
Yes	37.2	549	80.6	
**Household source of earnings**				0.040
Farming	60.8	898	74.8	
Wages	24.3	359	80.5	
Remittances	14.9	220	71.2	
Total	100.0	1478	75.7	

### Association between access to healthcare, demographic and socio-economic factors

From Table 2, results of the cross tabulations show that all demographic and socio-economic factors with the exception of gender, region, residence, household headship, education, religion and land ownership, were significantly associated with access to healthcare (age group, living alone, marital status, poverty status, bicycle ownership, and source of earnings).

More than three quarters accessed healthcare in the last 30 days preceding the survey. Access to healthcare was higher among older persons who were younger, lived with other people, and were married. In addition, older persons’ access to healthcare was higher among those who were from non-poor households, owned bicycle, and depended on wages as regular source of earnings.

### Distribution of sick older persons by health need and disability factors

From Table 3, 44% of the respondents were sick for 15 or more days and 45% missed work due to illness for 1 to 7 days. About a quarter (23%) had ever smoked and more than a quarter (28%) reported at least one NCD (heart disease, hypertension and diabetes). Disability was more pronounced with sight (46%) followed by walking (36%), hearing (20%) and memory (19%). Few sick older persons had some difficulty with self-care (9%) and communication (6%). Overall, four in ten (42%) sick older persons had some difficulty in at least one of the functional domains.

**Table 3:** **Distribution of sick older persons by health need and disability factors and access to healthcare in Uganda**

**Health need variables**	**Percent (%)**	**Frequency**	**% Accessed healthcare**	**p-value**
**Number of days ill**				0.000
1-7 days	37.8	558	82.0	
8-14 days	18.7	276	83.2	
15+ days	43.6	644	67.0	
**Days missed work due to sickness**				0.000
None	24.8	366	69.1	
1-7 days	44.5	657	81.0	
8-14 days	11.8	175	80.2	
15+ days	18.9	280	68.9	
**Ever smoked/smokes**				0.270
No	76.7	1134	76.4	
Yes	23.3	344	73.4	
**Reported an NCD (HBP, diabetes, heart disease)**				0.045
No	72.1	1065	74.2	
Yes	27.9	413	79.6	
**Has difficulty seeing even if wearing glasses**				0.025
None	53.2	786	78.4	
Some	35.4	523	73.6	
A lot/can’t	11.4	168	69.5	
**Has difficulty hearing even if wearing hearing aid**				0.135
None	79.9	1181	76.8	
Some	17.1	253	72.2	
A lot/can’t	3.0	44	66.9	
**Difficulty walking or climbing steps**				0.000
None	63.6	940	80.4	
Some	23.8	351	70.2	
A lot/can’t	12.6	187	62.5	
**Difficulty remembering or concentrating**				0.000
None	80.9	1196	77.4	
Some	14.7	218	72.2	
A lot/can’t	4.3	64	54.7	
**Difficulty with self-care - bathing, dressing, feeding**				0.001
None	90.7	1340	77.0	
Some	6.8	100	65.3	
A lot/can’t	2.5	37	55.0	
**Difficulty communicating - understanding others**				0.013
None	94.3	1394	76.3	
Some	4.3	64	70.1	
A lot /can’t	1.4	20	48.8	
**Aggregate disability status**				0.000
Not disabled	58.4	863	80.0	
Disabled	41.6	615	69.6	
**Total**	**100.0**	**1478**	**75.7**	

### Association between access to healthcare and health need and disability factors

From Table 3, results of the cross tabulations show that all health need and disability factors with the exception of smoking and hearing difficulty were significantly associated with access to healthcare (duration of illness, days missed work, self-reported NCDs, sight difficulties, walking difficulties, remembering difficulty, self-care limitations, communications difficulties and overall disability).

Access to healthcare was higher among older persons who were sick for 8–14 days (83%), missed work for 8–14 days (81%), and reported at least one NCD (80%). In addition, older persons’ access to healthcare was lower among those who had a lot of sight difficulties (70%), walking difficulties (63%), and memory problems (55%). In addition, those who had self-care challenges (55%) and communication problems (49%) had reduced access to healthcare. Overall, access to healthcare was lower for those who were disabled (70%).

### Reasons for not accessing healthcare

Of the 1,478 respondents who needed healthcare, 359 (24%) did not access it for the following reasons: 145 (40%) used informal care, 50 (14%) considered the illness to be mild, 62 (17%) did not have access to health facilities, and 80 (22%) reported financial barriers. In addition, 23 (6%) reported unavailability of quality of care.

### Multivariable results

From Table 4, three models were fit to measure the relationship between healthcare access in the last 30 days and selected explanatory variables. The models excluded variables that were not significant (p > 0.05) at the bivariate level of analysis. In model 1, we modeled healthcare access with predisposing (demographic and socio-economic) factors. The only variable that had a significant relationship with healthcare access was age group. Older persons who were age 70–79 *(RR = 0.90, 95*% *CI: 0.83-0.98)* and age 80+ *(RR = 0.80, 95*% *CI: 0.69-0.93)*, had reduced access to healthcare, in comparison to those age 50–59.

**Table 4:** **Adjusted incidence risk ratios for regression of access to healthcare in last 30 days on selected explanatory variables, among older persons in Uganda**

	**Model (1)**		**Model (2)**		**Model (3)**	
**Variables**	**Adjusted RR**	**95%** **CI**	**Adjusted RR**	**95%** **CI**	**Adjusted RR**	**95%** **CI**
**Age group**						
50-59	1.00		1.00		1.00	
60-69	1.03	[0.96-1.10]	1.04	[0.97-1.11]	1.05	[0.98-1.12]
70-79	0.90^*^	[0.83-0.98]	0.91^*^	[0.84-1.00]	0.95	[0.87-1.03]
80+	0.80^**^	[0.69-0.93]	0.82^**^	[0.71-0.95]	0.87	[0.76-1.01]
**Living alone**						
Yes	0.89	[0.77-1.02]	0.89	[0.77-1.02]	0.90	[0.79-1.03]
No	1.00		1.00		1.00	
**Marital status**						
Married	1.00		1.00		1.00	
Divorced/separated	0.96	[0.86-1.08]	0.97	[0.86-1.09]	0.97	[0.87-1.09]
Widowed	0.97	[0.91-1.04]	0.99	[0.92-1.06]	0.98	[0.91-1.05]
**Poverty status**						
Poor			0.90^*^	[0.83-0.98]	0.91^*^	[0.83-0.99]
Not-poor			1.00		1.00	
**Household owns bicycle at present**						
Yes			1.07^*^	[1.00-1.14]	1.06	[0.99-1.13]
No			1.00		1.00	
**Household source of earnings**						
Farming			1.00		1.00	
Wages			1.08^*^	[1.01-1.16]	1.08^*^	[1.00-1.15]
Remittances			1.00	[0.90-1.11]	1.00	[0.91-1.11]
**Days did not work due to sickness**						
0					1.00	
1-7					1.19^***^	[1.10-1.30]
8-14					1.19^***^	[1.07-1.31]
15+					1.08	[0.97-1.21]
**Reported NCD++**						
Yes					1.09^*^	[1.01-1.16]
No					1.00	
**Difficulty walking**						
No					1.00	
Some					0.90^**^	[0.83-0.97]
A lot/can’t					0.84^**^	[0.75-0.95]
Observations	1602		1602		1602	

RR = Risk Ratios; CI = Confidence Interval; ^*^p < 0.05, ^**^p < 0.01, ^***^p < 0.001; ++ NCDs included hypertension- diabetes & heart disease; Model (1) = adjusting for predisposing factors; Model (2) = adjusting for enabling factors; Model (3) = adjusting for health need factors.

In model 2, we adjusted for enabling factors to the first model. Four variables were significantly associated with healthcare access: age group, household poverty status, ownership of bicycle as a means of transport and household major source of earnings. Older age (70+) and household poverty were associated with reduced access to healthcare in the last 30 days for older persons (see Table 4). On the other hand, household ownership of a bicycle (RR = 1.07, 95% CI: 1.00-1.14) and earning of wages (RR = 1.08, 95% CI: 1.01-1.16) increased access to healthcare in the last 30 days.

In the final model (model 3), we adjusted for health need and physical disability variables. In this model, physical disability eliminated the effect of age. Healthcare access in the last 30 days was significantly associated with household poverty status, household earnings, number of days missed work due to illness (severity of illness), self-reported NCDs and walking difficulty. Older persons from poor households (RR = 0.91, 95% CI: 0.83-0.99 had reduced access to healthcare in the last 30 days, in comparison to those from non-poor ones. In contrast, older persons who earned wages (RR = 1.08, 95% CI: 1.00-1.15) had more access to healthcare in the last 30 days, than those who depended on farming.

Older persons who missed work due to illness for 1–7 days (RR = 1.19, 95% CI: 1.10-1.30); and 8–14 days (RR = 1.19, 95% CI: 1.07-1.31) had increased access to healthcare in the last 30 days, in comparison to those who never missed a day. In addition, those who reported non-communicable diseases (NCDs) such as heart disease, hypertension or diabetes (RR = 1.09, 95% CI: 1.01-1.16); were more likely to access healthcare during the last 30 days, in comparison to those who did not report any. Finally, older persons who reported some walking difficulty (RR = 0.90, 95% CI: 0.83-0.97); or a lot of walking difficulty (RR = 0.84, 95% CI: 0.75-0.95) were less likely to access healthcare, compared to those who had none (see Table 4).

## Discussion

This study aimed at discussing the determinants of access to healthcare in the last 30 days among older persons in Uganda. The factors that were significantly associated with access to healthcare were household wealth status and earnings, severity of illness in terms of inability to work, self-reported NCDs and physical disability.

Household poverty limited older persons’ access to healthcare in Uganda. On the contrary, earning wages increased access to healthcare in the last 30 days for older persons. Household income has been reported as a significant enabling factor for access to healthcare both in developed [39-42] and developing countries like Brazil [43]. In developing countries, the available evidence indicates that access to healthcare is pro-rich households for example in Ghana [44], India [45], Hong Kong [46] and China [47]. Poverty reduces the affordability of the healthcare services, irrespective of their availability and greater health need [46-48]. In Brazil, low socio-economic status was associated with poor health status and limited access to healthcare [43].

Health need factors (severity of illness, NCDs and physical disability) were the most important determinants of access to healthcare among older persons in Uganda. Severity of illness or inability to work was associated with increased access to healthcare. This confirmed the argument that people seek for treatment for severe illnesses and life threatening conditions. A study in the US based on national survey data from the non-institutionalized US individuals older than 50 years from the 2006 and 2008 waves of the Health and Retirement Study reported that, older people with worsening health were more likely to use healthcare services [49].

Older persons with NCDs (hypertension, heart disease and diabetes) used healthcare more than those without. Chronic health conditions create greater need for healthcare. The discourse on NCDs and access to healthcare has mixed findings. In some studies, NCDs have been associated with increased healthcare utilization among older persons, for example, in Hong Kong [46], Singapore [50], in rural South Africa [51] and Mexico [52]. Similar findings have been reported in some developed countries such as Spain [53]. However, in some developing countries, older persons with NCDs have limited utilization of healthcare for example in India [45], China [47] and Hong Kong [46].

Physical disability significantly reduced access to healthcare among older persons. Several mechanisms account for this disparity. First, mobility limitations decrease physical access to health facilities [54,55]. Second, disability leads to discrimination in access to healthcare for older persons [56]. Indeed, physical disability has been associated with poor health outcomes [57]; difficulties in accessing preventive and screening services [58]; among older persons in the US [59]. In Brazil, older women with limitations in instrumental activities of daily living had decreased likelihood of accessing pap-testing services [60]. There is only one study among black urban elderly in the US in the 1983, which reported that older people with physical disabilities had increased access to healthcare. But this was attributed to health and social programs that were instituted in the 1960’s targeting people with limited resources [61]. Older persons with mobility limitations face significant challenges in accessing healthcare services during their lifetime.

### Strength and limitations of the data

The strength of this paper was the use of a nationally representative sample of older persons from Uganda to investigate factors associated with access to healthcare among older persons. Reporting about access to healthcare within the last 30 days preceding the survey minimized recall bias.

Nonetheless, the limitations connected with the data included the following. First, there is a possibility of under-reporting of NCDs (heart disease, diabetes and hypertension) due to subjectivity of the self-reported measures – not based on diagnostic tests. The prevalence of NCDs (hypertension, diabetes and heart disease) could be lower than the actual prevalence in the older population in Uganda. Under-reporting was observed in Agincourt study in South Africa where self-reported hypertension was lower than the diagnostic test for the same condition [51]. Low income and education level limit the ability to self-report on certain health conditions [52]. Since majority of older persons had primary education, they could have had challenges on reporting about NCDs.

Second, we used cross-sectional data, which cannot bring out causality relationships between NCDs and access to healthcare well. The direction of causality between NCDs and access to healthcare is not clear [52]. Inability of cross sectional data to inform about quality of care has been a concern elsewhere [52]. In addition, the question on place of healthcare consultation restricted respondents to answer one choice or answer. From the data, it is not possible to ascertain any chronological health seeking behavior or multiple healthcare seeking options.

Third, the type and quality of care could not be ascertained from the data. Access to healthcare was measured by the question on consulting with healthcare provider. The data cannot tell whether general practitioners or specialized doctors were consulted. Furthermore, the data does not indicate whether the older persons obtained required dosage of drugs.

Notwithstanding these limitations, the paper provides evidence on factors associated with older persons’ access to healthcare in Uganda. These findings are important for policy makers, programme implementers and researchers.

## Conclusions

In the Ugandan context, health need factors (self-reported NCDs, severity of illness and mobility limitations) were the most important determinants of accessing healthcare in the last 30 days among older persons. In addition, enabling factors in general, and household wealth status and earnings in particular, were associated with access to healthcare. Poor older persons were less likely to access healthcare in the last 30 days. These findings have the following implications:

The Ministry of Gender, Labour and Social Development should scale up the measures for reducing poverty among older persons. Poverty was associated with limited access to healthcare. The Ministry of Gender, Labour and Social Development, should roll out the SAGE programme beyond the 14 pilot districts. The SAGE grants have enabled older persons to afford basic healthcare. Some studies have recommended promoting the extended family support system in cultures where it previously existed.

The Ministry of Health should strengthen the primary healthcare system in Uganda to provide long-term care to older persons with chronic NCDs such as diabetes, heart disease and hypertension. This is a critical intervention because older persons with NCDs, have greater need for healthcare. This recommendation has been made for rural South Africa [51]. Indeed, older persons need both preventive and curative healthcare and specialized services from geriatricians and gerontologists. The Ministry of Health needs to train these specialized cadres to be able to manage NCDs in the healthcare system in Uganda.

There is need to promote access to healthcare for older persons with physical disabilities [62]. Mobility limitations aggravate older persons’ challenge in access to healthcare, besides their age. The Ministry of Health could promote community outreach interventions or homecare services targeting disabled older persons. Finally, it is important to conduct further research to explore older persons’ healthcare access inequalities using either panel data or qualitative methods of inquiry.

## Additional file

Additional file 1:
**UNHS 2010 Questionnaire – health section showing measurement of outcome variable.**
