# Supplementary material for: Determinants of access to healthcare by older persons in Uganda: a cross-sectional study
Source: Int J Equity Health. 2015 Mar 7;14:26. doi: 10.1186/s12939-015-0157-z (PMC4354736; doi:10.1186/s12939-015-0157-z)
Supplement: Additional file 1: — UNHS 2010 Questionnaire – health section showing measurement of outcome variable. [file 12939_2015_157_MOESM1_ESM.pdf]

Section 5: Health: All Household Members

Ask the following questions about all members of the household **(usual and regular)**.

P E R S O N I D	During the past 30 days, did you suffer from any illness or injury?<br><br>1= Yes<br>2= No **(>> 14)**	For how many days did you suffer due to illness or injury during the past 30 days?<br><br>**Days**	For how many days did you have to stop doing your usual activities due to illness or injury during the past 30 days?<br><br>**Days**	Can you describe the **major** symptoms of the illness or injury that you **primarily** suffered from during the past 30 days?<br><br>**Record up to 2 symptom codes**<br><br>**See codes for Col. 5 below**		Was anyone consulted (e.g. a doctor, nurse, pharmacist or traditional healer) for the major illness or injury during the past 30 days?<br><br>1= Yes **(>> 8)**<br>2= No	Why was no one consulted for the major illness?<br><br>See code below<br><br>**[>> 14]**	Where did you go for the first consultation during the past 30 days?<br><br>1= Drugs at Home **(>> 14)**<br>2= Neighbor/Friend<br>3= Community health worker<br>4= HOMAPAK drug distributor<br>5= Ordinary shop<br>6= Drug shop/Pharmacy<br>7= Private clinic<br>8= Health unit government<br>9= Health unit NGO<br>10= Hospital government<br>11= Hospital NGO<br>12= Traditional healer<br>96= Other (specify)	Distance to the place where this treatment was sought for in km?	What was the cost of this consultation, including any medicine prescribed even if purchased elsewhere?<br><br>**UG. SHS**
**1**	**2**	**3**	**4**	**5a**	**5b**	**6**	**7**	**8**	**9**	**10**

Codes for Column 5

1= Diarrhoea (acute)
2= Diarrhoea (chronic, 1 month or more)
3= Weight loss (major)
4= Fever (acute)
5= Fever (recurring)
6= Malaria
7= Skin rash
8= Weakness
9= Severe headache
10= Fainting
11= Chills (feeling hot and cold)
12= Vomiting
13= Cough
14= Coughing blood
15= Pain on passing urine
16= Genital sores
17= Mental disorder
20= Abdominal pain
21= Sore throat
22= Difficulty breathing
23= Burn
24= Fracture
25= Wound
26= Child birth related
96= Other (specify)

Codes for Column 7

1= Illness mild
2= Facility too far
3= Hard to get to facility
4= Too dangerous to go
5= Available facilities are costly
6= No qualified staff present
7= Staff attitude not good
8= Too busy / long waiting time
